# Optimizing literature search in systematic reviews – are MEDLINE, EMBASE and CENTRAL enough for identifying effect studies within the area of musculoskeletal disorders?

**DOI:** 10.1186/s12874-016-0264-6

**Published:** 2016-11-22

**Authors:** Thomas Aagaard, Hans Lund, Carsten Juhl

**Affiliations:** 1 Department of Physiotherapy, Holbaek University Hospital, Holbaek, Denmark; 2 Research Unit for Musculoskeletal Function and Physiotherapy, Institute for Sports Science and Clinical Biomechanics, University of Southern Denmark, Odense, Denmark; 3 Center for Evidence-based practice, Bergen University College, Bergen, Norway; 4 Department of Rehabilitation, Copenhagen University Hospital, Herlev, Gentofte, Denmark

**Keywords:** Information retrieval, Bibliometric, MECIR guidelines, Evidence-based medicine, Relative recall, Literature searching, Systematic review, Musculoskeletal area

## Abstract

**Background:**

When conducting systematic reviews, it is essential to perform a comprehensive literature search to identify all published studies relevant to the specific research question. The Cochrane Collaborations Methodological Expectations of Cochrane Intervention Reviews (MECIR) guidelines state that searching MEDLINE, EMBASE and CENTRAL should be considered mandatory. The aim of this study was to evaluate the MECIR recommendations to use MEDLINE, EMBASE and CENTRAL combined, and examine the yield of using these to find randomized controlled trials (RCTs) within the area of musculoskeletal disorders.

**Methods:**

Data sources were systematic reviews published by the Cochrane Musculoskeletal Review Group, including at least five RCTs, reporting a search history, searching MEDLINE, EMBASE, CENTRAL, and adding reference- and hand-searching. Additional databases were deemed eligible if they indexed RCTs, were in English and used in more than three of the systematic reviews. Relative recall was calculated as the number of studies identified by the literature search divided by the number of eligible studies i.e. included studies in the individual systematic reviews. Finally, cumulative median recall was calculated for MEDLINE, EMBASE and CENTRAL combined followed by the databases yielding additional studies.

**Results:**

Deemed eligible was twenty-three systematic reviews and the databases included other than MEDLINE, EMBASE and CENTRAL was AMED, CINAHL, HealthSTAR, MANTIS, OT-Seeker, PEDro, PsychINFO, SCOPUS, SportDISCUS and Web of Science. Cumulative median recall for combined searching in MEDLINE, EMBASE and CENTRAL was 88.9% and increased to 90.9% when adding 10 additional databases.

**Conclusion:**

Searching MEDLINE, EMBASE and CENTRAL was not sufficient for identifying all effect studies on musculoskeletal disorders, but additional ten databases did only increase the median recall by 2%. It is possible that searching databases is not sufficient to identify all relevant references, and that reviewers must rely upon additional sources in their literature search. However further research is needed.

## Background

Systematic reviews (SR) and meta-analyses (MA) are key elements in both evidence-based healthcare [1] and evidence-based research [2] By synthesizing the available evidence, SRs support clinicians in making well-informed decisions about health care [3] and researchers in deciding which topics are the most relevant for new research [4]. When conducting SRs, it is essential to perform a comprehensive literature search to identify all published studies relevant to the research question as a failure to do so can result in selection bias and distort the conclusion of the review by potentially over- or underestimating of the treatment effect [3, 5]. One of the recommended methods to identify scientific literature in health science is searching electronic databases [3, 6]. However, when doing a high quality search two main questions arise; which databases is necessary to searched, and how many? According to the Cochrane Collaborations Methodological Expectations of Cochrane Intervention Reviews (MECIR) three main databases: MEDLINE, EMBASE and CENTRAL are mandatory electronic databases to search when performing a Cochrane Review [7]. Several studies indicate that searching MEDLINE identifies the highest number of studies [8–10] and others that the gains from searching beyond MEDLINE and in particular EMBASE are modest [11], however multiple studies have found that searching MEDLINE alone is not sufficient [8–10, 12–19]. In addition, when analysing the use of databases in Cochrane reviews, it was found that between 1 and 27 different databases was used [20], even though some studies indicates that searching no more than 3–5 databases seems to be sufficient,[8, 16, 17] and searching only one database would not be enough [19]. Even though MEDLINE, EMBASE and other major medical databases yield a high proportion of relevant studies, some studies found it necessary to include other sources such as reference- and citation search, browsing conference proceedings, asking experts and alike to identify all the relevant studies [9, 14, 17, 19]. The difference between the results from these studies could be due to their evaluation of different areas or due to the methods used to search the different databases; hence some of the above mentioned studies construct a new search strategy to identify studies in a given area thereby making the evaluation be dependent on not just the database, but also the quality/accuracy of the search strategy constructed [21–23]. The great variations thus indicate a need to evaluate if the MECIR guideline recommendations to search MEDLINE, EMBASE and CENTRAL combined would be enough when performing a literature search or whether additional databases should be added to this list.

In order to focus this evaluation, we choose to concentrate on musculoskeletal disorders. The area of musculoskeletal injuries and diseases is the leading cause of long-term pain and physical disability [24–26] and are associated with 130 million health care encounters and estimated to cost over $50 billion annually in the United States [27]. In addition, the Cochrane Musculoskeletal Review Group (CMSG) is among the largest review groups in the Cochrane Collaboration, responsible for more than 200 SRs.

The aim of this study is therefore to evaluate the relative recall in the databases recommended by MECIR for systematic literature searches within the area of musculoskeletal disorders. Furthermore, this study addressed the question: What is the increase in recall when searching additional databases to searching MEDLINE, EMBASE and CENTRAL combined?

## Methods

### Selection of systematic reviews

All SRs from the Cochrane Database of Systematic Reviews (CDSR) published by CMSG were obtained [28]. SRs were excluded if they: (i) did not include at least five randomised controlled trials (RCT), as the consequence of missing one study in reviews with few studies included would affect the overall percentage more than with a higher total of included studies. (ii) had been withdrawn, (ii) did not report any search history in the SR (iii) did not search all of the following sources: MEDLINE, EMBASE, CENTRAL, reference- and hand searching, as recommended by The Cochrane Handbook [3] and MECIR guidelines [7]. This strategy was defined in order to identify systematic reviews, which had included all (or close to all) relevant studies related to at certain research question by both searching electronic databases and other sources.

The recall was used to evaluate the ability of a search strategy to identify all relevant studies [29]. Recall can be defined as the percentage of relevant records retrieved divided by the total number of included studies in the individual systematic reviews. However, to estimate true recall one need to know the total amount of relevant records in a database, which is not an easy (if not impossible) task. Thus often, relative recall is estimated by firstly defining a pool of relevant records (the included studies in a SR) and then determines what proportion of this pool the literature search retrieves.[30, 31] In this study we therefore used the included studies in each of the included SRs as the pool of relevant records.

### Identification of databases

From the pool of SRs, a list of databases used was created. Databases were ranked in descending order according to how many SRs that had searched the database. Databases other than MEDLINE, EMBASE and CENTRAL were deemed eligible if they (i) was indexing RCTs, (ii) was in English, (iii) was used by at least three SRs.

### Data-extraction

The following data were extracted for each included SR: (i) details of the search strategy as described in the review (ii) date of when the search was performed or updated (iii) full bibliographic details of all primary studies included in the SR (i.e. title of the study, author names, journal title, publications year etc.).

### Searching individual databases

The search strategy/strategies reported in each SR was replicated and used for searching all the databases included in the SR. For databases with no reported search strategy, the MEDLINE search strategy was replicated and searched in all included databases. MEDLINE syntax (i.e. fields, truncation, adjacency) was modified to the individual database. When possible, the exact search dates from the SR was used for each database. However, CENTRAL for instance, only allows specification by month and year. End Note ×7.5.3 software (Thomson Reuters™) was used to manage records retrieved from searches of electronic databases.

### Statistical analysis

Relative recall for each of the included database was calculated separately and for each of the included SR. Relative median recall for each database was calculated for all included SRs combined. Cumulative median relative recall was estimated for searches in MEDLINE, EMBASE and CENTRAL adding databases in descending order (based on how often the databases was searched in the SRs published by CMSG). Data managing was performed using Microsoft Excel 2016 and data analysis was performed using STATA version 13.1 (StataCorp, College Station, Texas) software package.

### Additional analyses

Subgroup analysis was pre-specified and planned to assess the cumulative median recall for subgroups, *rehabilitation*, *medicine* or *other content* (surgery, lifestyle intervention, electrical stimulation etc.) as previous studies have found differences in recommendations depending on the topics searched [32, 33]. One post-hoc sensitivity analysis was conducted to address to what extend the inclusion of SRs with a cut-off of three included RCTs instead of five would affect the result.

## Results

### Eligible databases and systematic reviews

**Fig. 1:**
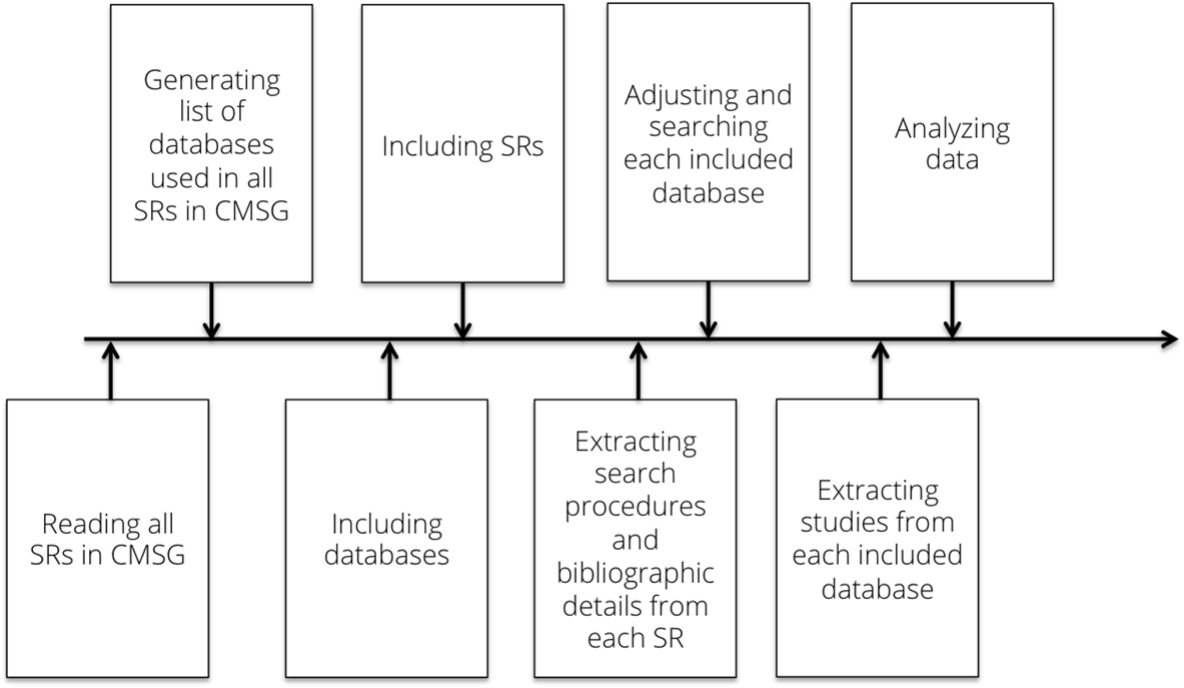
A timeline of the selecting, inclusion and analysis process

**Fig. 2:**
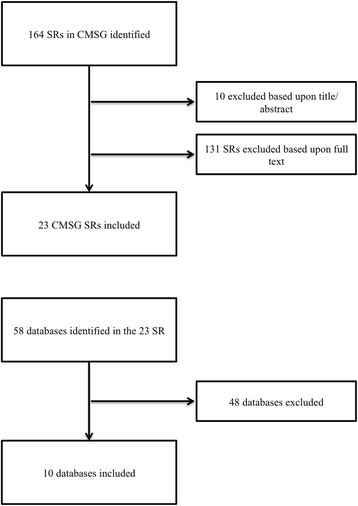
Flowchart for inclusion of Cochrane reviews and databases

A timeline is displayed in Fig. 1. A set of 164 SRs where identified and obtained from the CMSG on March 3, 2013 and revisited for an update on July 3 2013 by the first author (Fig. 2). Of the 164 SRs assessed for eligibility by title and abstract, 10 were excluded. Nine for being withdrawn and one for being an overview of reviews. Of the remaining 154 SRs assessed in full-text, 114 were excluded, as they did not search one or more of the following sources: MEDLINE-, CENTRAL-, EMBASE, or reference- and hand searching. 11 SRs were excluded, as they did not include five or more studies in their analysis. Six were excluded for not reporting any search strategy in the SR, neither reporting where one could be acquired. A final set of 23 SRs finally met all inclusion criteria [34–56], (Table 1).

**Table 1:** Characteristics of the included Cochrane reviews

Author, year published (ref.)	Year assessed as up-to-date	Review title	Number of sources searched	Number of studies included	Reported search strategy	Assigned group
Adie et al., 2012 [34]	2012	Cryotherapy following total knee replacement (Review)	11	12	MEDLINE, CENTRAL, EMBASE, CINAHL, PEDro, Web of Science	Rehabilitation
Bartels et al. 2009 [35]	2007	Aquatic exercise for the treatment of knee and hip osteoarthritis (Review)	9	6	MEDLINE, EMBASE CINAHL, Web of Science, PEDro.	Rehabilitation
Bellamy et al. 2009 [36]	2006	Intraarticular corticosteroid for treatment of osteoarthritis of the knee (Review)	8	28	Standard search strategy	Medicine
Bellamy et al. 2009 [37]	2006	Viscosupplementation for the treatment of osteoarthritis of the knee (Review)	8	103	Standard search strategy	Medicine
Coghlan et al. 2009 [38]	2008	Surgery for rotator cuff disease (Review)	8	14	Standard search strategy	Other content
Colebatch et al. 2011 [39]	2011	Safety of non-steroidal anti-inflammatory drugs, including aspirin and paracetamol (acetaminophen) in people receiving methotrexate for inflammatory arthritis (rheumatoid arthritis, ankylosing spondylitis, psoriatic arthritis, other spondyloarthritis) (Review)	8	17	MEDLINE, EMBASE, CENTRAL.	Medicine
Cranney et al. 2010 [40]	2000	Calcitonin for preventing and treating corticosteroid-induced osteoporosis (Review)	6	9	MEDLINE	Medicine
Fidelix et al. 2009 [41]	2006	Diacerein for osteoarthritis (Review)	7	7	Standard search strategy	Medicine
Karjalainen et el. 2009 [42]	2000	Multidisciplinary rehabilitation for fibromyalgia and musculoskeletal pain in working age adults (Review)	8	10	MEDLINE	Rehabilitation
Katchamart et al. 2010 [43]	2010	Methotrexate monotherapy versus methotrexate combination therapy with non-biologic disease modifying anti- rheumatic drugs for rheumatoid arthritis (Review)	5	20	MEDLINE, EMBASE, CENTRAL,	Medicine
Khan et al. 2009 [44]	2008	Multidisciplinary rehabilitation programmes following joint replacement at the hip and knee in chronic arthropathy (Review)	10	5	MEDLINE	Rehabilitation
Lethaby et al. 2013 [45]	2013	Etanercept for the treatment of rheumatoid arthritis (Review)	14	9	MEDLINE	Medicine
de Morton et al. 2009 [46]	2007	Exercise for acutely hospitalised older medical patients (Review)	11	10	MEDLINE	Rehabilitation
Nuesch et al. 2010 [47]	2009	Oral or transdermal opioids for osteoarthritis of the knee or hip (Review)	11	10	MEDLINE, EMBASE, CINAHL, CENTRAL.	Medicine
Osiri et al. 2010 [48]	2003	Leflunomide for the treatment of rheumatoid arthritis (Review)	8	33	MEDLINE	Medicine
Richards et al. 2011 [49]	2011	Antidepressants for pain management in rheumatoid arthritis (Review)	6	8	MEDLINE, EMBASE	Medicine
Richards et al. 2012 [50]	2012	Muscle relaxants for pain management in rheumatoid arthritis (Review)	6	6	MEDLINE, EMBASE	Medicine
Ruiz Garcia et al. 2011 [51]	2011	Certolizumab pegol (CDP870) for rheumatoid arthritis in adults (Review)	14	6	MEDLINE, EMBASE, CINAHL, CENTRAL, SCOPUS, TOXLINE, Web of Science.	Medicine
Rutjes et al. 2010 [52]	2010	Therapeutic ultrasound for osteoarthritis of the knee or hip (Review)	14	5	MEDLINE, EMBASE, CINAHL, CENTRAL, PEDro.	Other content
Rutjes et al. 2010 [53]	2009	Transcutaneous electrostimulation for osteoarthritis of the knee (Review)	13	18	MEDLINE, EMBASE, CINAHL, CENTRAL, PEDro.	Other content
Wajon et al. 2009 [54]	2005	Surgery for thumb (trapeziometacarpal joint) osteoarthritis (Review)	7	11	MEDLINE, CENTRAL, EMBASE, CINAHL, AMED.	Other content
Whittle et al. 2011 [55]	2011	Opioid therapy for treating rheumatoid arthritis pain (Review)	5	11	MEDLINE, EMBASE, CENTRAL	Medicine
Winzenberg et al. 2010 [56]	2010	Vitamin D supplementation for improving bone mineral density in children (Review)	8	7	MEDLINE.	Medicine

The generated list of databases other than MEDLINE, EMBASE and CENTRAL included a total of 58 databases identified in the 23 included SRs. Of these 58 databases, 48 databases were excluded; 10 did not index RCTs (i.e. trial registry etc.), nine where included in other databases (i.e. Premedline in MEDLINE etc.), 28 were used in less than three SRs, and one database was not in English. The following 10 databases met the inclusion criteria: AMED (via EBSCOhost), CINAHL (via EBSCOhost), HealthSTAR (via OVID), MANTIS (via OVID), OT-Seeker, PEDro, PsychINFO (via OVID), SCOPUS, SportDISCUS (via EBSCOhost) and Web of Science. Searching MEDLINE was performed using the host specified in the SR (i.e. via OVID or Pubmed), EMBASE via the OVID and CENTRAL via the Wiley InterScience portal.

### Characteristics of the included systematic reviews

The 23 SRs included a total of 365 primary studies. Each review included from 5 to 103 studies (median 10) (Table 1). The number of search strategies reported in the SRs ranged from 1 to 7 (median 2). Eleven out of 23 SRs reported only one search strategy; 4 of which reported a “standard search strategy” that was adapted to other databases searched, while 7 reported the search strategy used for MEDLINE (Table 1). Of the 23 SRs, the intervention in 5 was classified as *rehabilitation*, 14 as *medicine* and 4 was classified as *other content* (Table 1).

### Synthesis of results

Table 2 displays the median relative recall for the combined search in MEDLINE, EMBASE and CENTRAL and for the additional 10 databases included. Data shown are the median recall and interquartile range (IQR) from the total number of SRs and for the three subgroups separately. MEDLINE, EMBASE and CENTRAL combined yielded a median recall of 88.9% (IQR 81.6–100%), followed by SCOPUS (85.7%) and HealthSTAR (83.3%) (Table 2).

**Table 2:** Median recall analysis for each database in descending order

No. in descending order	Databases	Median (IQR)
1–3	MEDLINE + EMBASE + CENTRAL	88.9% (81.6–100%)
4	SCOPUS	85.7% (61.8–100%)
5	HealthSTAR	83.3% (62.4–89.4%)
6	Web of Science	55.6% (1.8–79.3%)
7	CINAHL	14.3% (1.9–40%)
8	MANTIS	12.5% (2.9–16.6%)
9	PEdro	0% (0–20.7%)
10	SportDISCUS	0% (0–9.1%)
11	AMED	0% (0–7.7%)
12	PsychINFO	0% (0–0%)
13	OT-seeker	0% (0–0%)

**Fig. 3:**
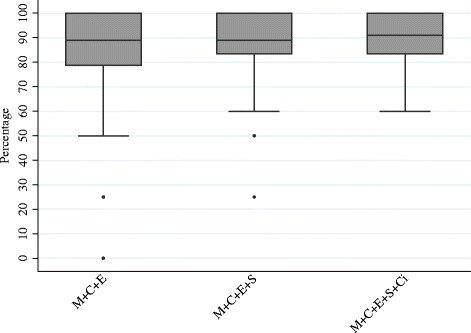
The accumulating percentage as a boxplot

Results of the overall cumulative median analysis on relative recall are displayed in Fig. 3 and Table 3. The most exhaustive search (i.e. the minimum number of databases required to be searched to retrieve the maximum number of studies) involved searching MEDLINE, EMBASE and CENTRAL with the addition of SCOPUS and CINAHL. Results show that adding these databases to MEDLINE, EMBASE and CENTRAL increased the median recall by 2.0 percentage points, from 88.9% to a median recall of 90.9% (IQR 83.3–100%). Adding the remaining 8 databases did not increase the median recall.

**Table 3:** Overall cumulative median analysis on relative recall

Combination of databases	Median recall (IQR)
M + E + C	88.9% (81.1–100%)
M + E + C + S	88.9% (83.3–100%)
M + E + C + S + Ci	90.9% (83.3–100%)

*Abbreviations*: *M* + *E* + *C* MEDLINE + EMBASE + CENTRAL, *M* + *E* + *C* + *S* MEDLINE + EMBASE + CENTRAL + SCOPUS, *M* + *E* + *C* + *S* + *Ci* MEDLINE + EMBASE + CENTRAL + SCOPUS+ Cinahl

### Additional analyses

#### Subgroup analysis

Subgroup analyses according to content area demonstrated some variations. The most exhaustive search for the *rehabilitation* group involved searching MEDLINE, EMBASE and CENTRAL with the addition of SCOPUS and CINAHL for a cumulative median recall of 100% (IQR 60–100%) (Table 4), the *medicine* group with the addition of SCOPUS for a cumulative median recall of 87.3% (IQR 83.3–97.7%) (Table 4), and the *other content* groups with the addition of Scopus and CINAHL for a cumulative median recall of 100% (IQR 97.7–100%) (Table 4).

**Table 4:** Cumulative subgroup analysis according to content area

Subgroups	Combination of databases	Median (IQR)
Rehabilitation	M + E + C	83.3% (50–100%)
	M + E + C + S	83.3% (60–100%)
	M + E + C + S + Ci	100% (60–100%)
Medicine	M + E + C	87.3% (79.9–97.7%)
	M + E + C + S	87.3% (83.3–97.7%)
Other content	M + E + C	97.2% (93.6–100%)
	M + E + C + S	100% (97.7–100%)

*Abbreviations*: *M* + *E* + *C* MEDLINE + EMBASE + CENTRAL, *M* + *E* + *C* + *S* MEDLINE + EMBASE + CENTRAL + SCOPUS, *M* + *E* + *C* + *S* + *Ci* = MEDLINE + EMBASE + CENTRAL + SCOPUS + Cinahl

#### Post-hoc analysis

Post-hoc sensitivity analysis showed that with the inclusion of SRs with at least three included RCTs, 4 SRs would be added to the analyses [57–60]. The analyses showed that the cumulative median recall increased when adding these SRs, however the IQR remained unchanged (Table 5).

**Table 5:** Overall cumulative median analysis on relative recall with a cut-off of three included studies

Combination of databases	Median recall (IQR)
M + E + C	90.1% (81.1–100%)
M + E + C + S	90.1% (83.3–100%)
M + E + C + S + Ci	100% (84.5–100%)

*Abbreviations*: *M* + *E* + *C* MEDLINE + EMBASE + CENTRAL, *M* + *E* + *C* + *S* MEDLINE + EMBASE + CENTRAL + SCOPUS, *M* + *E* + *C* + *S* + *Ci* MEDLINE + EMBASE + CENTRAL + SCOPUS+ Cinahl

## Discussion

This study supports the recommendations by Cochrane Collaboration to prioritize MEDLINE, EMBASE and CENTRAL as the basic databases for literature search to locate RCTs in the musculoskeletal area. Secondly, this study indicates that besides MEDLINE, EMBASE and CENTRAL a literature search to locate RCTs in the musculoskeletal area could also consider SCOPUS and CINAHL. Finally, this study indicates that even with the addition search of 10 other often used databases median recall is not improved noticeably.

Thirteen different databases were not enough to identify all relevant references. Searching MEDLINE, EMBASE and CENTRAL retrieved 88.9%, and searches in 10 additional databases increased the median recall by only 2 percentage point. Thus, results from this study could be interpreted, as an indication that searching databases is not sufficient to identify all relevant references and that other sources must be included in the literature search in order to achieve a larger recall. This study does not evaluate which source that may be the most important. Savoie et al. and Helmer et al. [61, 62] found that 29.2% of all items retrieved for two SRs could be uncovered by extended systematic search methods; searching subject- specific or specialized databases (including trial registries), hand-searching, scanning reference lists, and communicating personally with experts. Yet Robinson et al. [63] recently showed that researchers do not cite all possible previous trials, and that less than half (38%) of RCTs could be identified by citation network searching.

It therefore remains to be evaluated how much higher recall could be achieved by supplementing the database search with reference and/or citation search, and which impact if any these additional sources have on the pooled estimate effect.

Searching SCOPUS and CINAHL increased the median recall by 2 point. Yet, as results from the subgroup analysis showed, each database contributed differently depending on the field groups searched. SCOPUS increased the median recall slightly in the *Other* group, and had some effect on the IQR in all three groups. This could be due to the fact that SCOPUS is a generic database containing studies from a wide range of subject fields. The large increase in median recall in the *rehabilitation* group searching CINAHL could be because CINAHL is a database including research from health care professionals often involved with rehabilitation. The fact that CINAHL only increased the recall in the area of rehabilitation are supported by Beckles et al. who strongly recommends that the database should be relegated too selective rather than routine searching due to a very low proportion of unique references [64].

Results from the post-hoc sensitivity analyses showed not surprisingly that the inclusion of studies with a low proportion of included studies could introduce high risk of bias of the results. Adding only four more studies increased the median recall to 100% and by 10% compared to the main results. Yet, as the IQR of the results are unchanged, this reinforce the notion, that searching additional databases is less likely to add more studies.

To our knowledge only one earlier study concluded that one database was enough in order to achieve full recall. Kelly et al. [65] concluded that MEDLINE was enough to identify all relevant studies for their specific question. However, they concluded that to fully capture the complete body of available literature on other subjects might require searching multiple databases [20]. This is strongly supported by a number of other studies [8–13, 15, 16, 18–22, 33, 66–78]. Studies evaluating this question within the musculoskeletal field make the same conclusion: searching more than one database is necessary [14, 17, 23, 32, 79–81]. Based on results from earlier studies and the results from the present study, recommendation for an optimal systematic literature search to locate RCTs within the musculoskeletal area may be to use the three generic databases: MEDLINE; EMBASE and CENTRAL, and an additional two or more other databases. However, this search should ad other sources such as reference- and citation search, grey literature, conference proceedings, and contact experts within the area as results from this study suggest that 10% could be missed when only searching electronical databases to identify relevant studies.

### Limitations of this study

This study has some limitations. An important limitation of this study and other studies evaluating the recall of a systematic literature search is the definition of the true number of studies that should be identified. In this study we defined this as the number of studies deemed relevant (i.e. included) in a SR, yet as a SR seek to answer a well-defined question, there are some limitations to whether the included SRs in this study fully represent the various areas of the musculoskeletal field. Another limitation of any study reproducing an original search strategy at a later date is that the contents and indexing of databases change over time, and not all of these changes can be rewound.

Another limitation to this study is the underlining assumption that the systematic literature search strategy used in each SR did capture all relevant studies in the database searched. The result from the present study does not evaluate this question, yet Sampson et al. [82] found that errors in electronic search strategies reduce the effectiveness of electronic search strategies. Further research is needed to evaluate not only the recall of studies retrieved using a search strategy, but also comparing this to the recall of studies indexed in a database by bibliographic verification: searching for known items [83], thereby addressing the key question, what is indexed in a database? and what is actually retrieved when searching this database?

The aim of this study was to evaluate the relative recall in the databases recommended by MECIR for systematic literature searches to locate RCTs within the area of musculoskeletal disorders. The use and limitations of the method to the musculoskeletal area thus clearly limits the general conclusion from this study. However, our results are in line with other studies evaluating literature search in electronic databases.

The strengths of this study lies in the systematic approach of selecting Cochrane SRs of the highest quality, and combining results of the literature search from these SRs in a way that make SRs with a high number of studies included equal to those with low number of studies included. This is also one of few studies [20, 23, 84] that have combined multiple databases using cumulative analysis, thereby accepting what researchers have urged in the past, that searching one database is not enough but investigating what a combined search would yield.

## Conclusions

Searching MEDLINE, EMBASE and CENTRAL is not sufficient for identifying all effect studies within the area of musculoskeletal disorders. Literature searches in ten additional databases only increases the median recall by 2 percentage point.

It remains to be evaluated how much higher the relative recall could be achieved by supplementing the database search with reference and citation search. Further studies where the same methods are applied on different content areas needs to be performed, to see if the assumption that the way to perform a search depends on the content area is true or not. It is possible that searching databases is not sufficient to identify all relevant references, and that reviewers must rely upon additional sources in their literature search, but further research on these additional sources is needed.

## Abbreviations

CDSR: Cochrane database of systematic reviews
CMSG: Cochrane musculoskeletal review group
IQR: Interquartile range
M + E + C: MEDLINE + EMBASE + CENTRAL
M + E + C + S: MEDLINE + EMBASE + CENTRAL + SCOPUS
M + E + C + S + Ci: MEDLINE + EMBASE + CENTRAL + SCOPUS+ Cinahl
MA: Meta-analyses
MECIR: Methodological expectations of cochrane intervention reviews
RCT: Randomised controlled trials
SR: Systematic reviews
